# What Drives Volunteers to Accept a Digital Platform That Supports NGO Projects?

**DOI:** 10.3389/fpsyg.2020.00429

**Published:** 2020-03-31

**Authors:** Jose Ramon Saura, Pedro Palos-Sanchez, Felix Velicia-Martin

**Affiliations:** ^1^Department of Business Economics, Rey Juan Carlos University, Madrid, Spain; ^2^Department of Financial Economy and Operations Management, University of Seville, Seville, Spain; ^3^Department of Business Administration and Marketing, University of Seville, Seville, Spain

**Keywords:** non-profit-making organization, Technology Acceptance Model, volunteers, website, social network

## Abstract

Technology has become the driving force for both economic and social change. However, the recruitment of volunteers into the projects of non-profit-making organizations (NGO) does not usually make much use of information and communication technology (ICT). Organizations in this sector should incorporate and use digital platforms in order to attract the most well-prepared and motivated young volunteers. The main aim of this paper is to use an extended Technology Acceptance Model (TAM) to analyze the acceptance of a technological platform that provides a point of contact for non-profit-making organizations and potential volunteers. The TAM is used to find the impact that this new recruitment tool for volunteers can have on an ever-evolving industry. The TAM has been extended with the *image and reputation* and *visual identity* variables in order to measure the influence of these non-profit-making organizations on the establishment and implementation of a social network recruitment platform. The data analyzed are from a sample of potential volunteers from non-profit-making organizations in Spain. A structural equation approach using partial least squares was used to evaluate the acceptance model. The results provide an important contribution to the literature about communication in digital environments by non-profit-making organizations as well as strategies to improve their digital reputation.

## Introduction

Over the past decade, the Internet has led to major changes in the organization of companies around the world (Lee and Kim, 2018). With the development of new technologies, corporations, institutions, and non-profit-making organizations have seen how communication strategies have changed. These changes consist of sending messages on new digital channels with which the company’s aims and goals are transmitted to users, volunteers, or anybody who is interested (Lee and Kim, 2018; Tam and Kim, 2019).

Social networks play an important role when changes happen quickly in an organization (Bojar, 1998), for example, with the changes and evolution of communication and marketing strategies (Nuri et al., 2019). On these digital platforms, users can give their opinions and generate content publicly, which is known as user-generated content (UGC) (Saura and Bennett, 2019; Saura et al., 2019a). Users can also interact with companies and organizations by writing comments, reviews, opinions, and criticisms (Abdelhamid et al., 2019).

This new two-way communication between users and organizations on the Internet (Aggelidis and Chatzoglou, 2019) has allowed non-profit-making organizations to implement and use communication strategies to convey positive feelings about their proposals and projects and to support social movements such as MeToo on the Internet (Mendes et al., 2018), World Environment Day (Reyes-Menendez et al., 2018), global warming (Jang and Hart, 2015), and others (Lau et al., 2019).

These new communication strategies using interaction with users have also led to changes in the non-profit-making organization sector as well as social institutions (Alonso-Cañadas et al., 2019). Although the non-profit-making sector has changed its medium and long-term strategies because of its social and non-economic goal, it is a sector that is increasing the use of new communication strategies with user interactions on digital channels (Iranzo and Farné, 2014). Variables such as the image, reputation, and visual identity of non-profit-making organizations using these channels allow them to be more easily identified and accepted, as well as permitting them to transmit their new ideas and social projects [(see initiatives Ecosia (Palos-Sanchez and Saura, 2018; Palos-Sanchez et al., 2018) and Lilo (Reyes-Menendez et al., 2018)] in order to increase the number of volunteers and users recruited to support their causes.

These facts have aroused the curiosity of researchers who have taken an interest in the study of the variables influencing the image of non-profit-making organizations (NGOs). These studies, as pointed out by Hasmath et al. (2019), analyze digital channels, such as social media profiles or web pages, in order to find the best way to define recruitment strategies. Non-profit-making organizations are now using digital channels to recruit new volunteers to support their projects and help the organizations achieve their goals (Aggelidis and Chatzoglou, 2019).

In this way, there have been changes in this sector, with different platforms that aim to attract volunteers who can support social projects, where volunteers can take part in one or more organizations using a single platform that promotes projects with similar goals (Guiang, 2017). It is important to note that this research focuses on the study of NGOs, non-governmental organizations that are independent of any government and usually are non-profit, and not on non-profit organizations (NPOs), which are businesses that have been granted tax-exempt status (Lloyd, 2005).

The main objective of this research paper is to analyze the technological acceptance of a social network that supports volunteer projects and that facilitates the contact between potential volunteers and non-profit-making organizations. To do this, the extended Technology Acceptance Model (TAM) was used with extended variables for the image, identity, and reputation of the organizations.

This research is original because of the importance given to the image, reputation, and visual identity variables of the organizations with an extended TAM to study the importance of variables that are influential for non-profit-making organizations in a digital ecosystem.

To this end, in this research, the results of a survey conducted with potential volunteers of non-profit organizations in Spain were studied, in order to understand how well the digital platform with which NGOs can recruit volunteer candidates is accepted. The study uses a structural equation approach with partial least squares (PLS) to evaluate the proposed acceptance model.

The research is organized in the following way. Firstly, the *Introduction* explains the paper. Then there is a *Literature Review*, which discusses relevant research in this area. Thirdly, the methodology and justification of the hypotheses are presented. The results are then analyzed. In the *Conclusions* section, discussions and conclusions are made in which the theoretical and practical implications of the research are given.

## Literature Review

Variables that affect users’ decisions to use a web platform have been studied on different occasions (Liu and Lee, 2010). For example, Kwon and Wen (2010) carried out an empirical study to observe user acceptance of social media and digital platforms in order to see if they offer services for promoting human relationships or simply to motivate users to use them with strategies to improve brand image or to maintain the brand reputation.

Using the evidence from previous research which showed the important influence of social networks on the reputation of NGOs, Gálvez-Rodríguez et al. (2016) analyzed the factors which influence the use of Twitter by NGOs in order to understand how information and news are communicated (Reyes-Menendez et al., 2019). They discovered that there is still room for improvement in these types of social ecosystem strategies in which an NGO’s goals and social actions form the volunteers’ image of them.

In order to investigate strategies for NGOs in continuously changing ecosystems, Iranzo and Farné (2014) studied NGOs’ behavior in environments driven by social movements using social networks such as Facebook or Twitter. The study showed the need for an improvement in the understanding of what drives NGO volunteers to express themselves on digital channels and how an NGO’s image influences volunteers who follow their actions on social media.

To understand NGOs as social causes that motivate volunteers who decide to support them, Lau et al. (2019) analyzed the motivating effect of people volunteering to support a social project and their ability to solve social problems. They concluded that volunteers should feel the need to express the purposes of NGOs with “likes” to show that they are proud of their actions, as well as to promote the image of the NGOs in terms of social, humanitarian, or environmental support. These results show the importance of volunteers having the feeling that they belong to an NGO so that that they share the same values.

In this changing ecosystem, in which the social identity of the users and their actions are shared on the Internet, investigating the reasons that drive NGOs to choose digital communication channels for their communication strategies are becoming increasingly important. For example, Hasmath et al. (2019) identified the behavior of NGOs in terms of the management decision-making for activities and social actions. They found that the new means of communication and transmission of information to volunteers needed to be studied so that messages were accepted and understood in a clear way, as at times, the noise generated by social activists who are against the initiatives of some NGOs can influence their reputation and therefore also the number of interested volunteers.

Although the term *volunteer* for an NGO does not usually lead to confusion, authors such as Gonzales et al. (2019) decided to extend the terms *volunteer* and *volunteer projects*. They center their attention on projects for voluntary purposes by providing data and scientific evidence to demonstrate the interest aroused in impulse users who want to become volunteers both in digital ecosystems and in traditional offline media. Their results coincided with the work by Vissers and Stolle (2014) about the new online and offline models of management in NGOs and political parties.

In similar studies, Gonzales et al. (2019) and Dickinger and Stangl (2013) showed interest in studying NGOs and their communication plans to convince volunteers to support their projects. In this way, Cheng (2020) analyzed the communication process between Internet users in the decision-making moment, such as making a purchase or joining an online community, so that messages are correctly transmitted and the objectives of the organization are achieved. The communication process pays special attention to the image of the organization, its visual identity, and its reputation (Dickinger and Stangl, 2013). Following Cheng’s results, Alonso-Cañadas et al. (2019) analyzed the organization, resource allocation, and policies of NGOs when managing social projects, by demonstrating the importance of image and reputation in order for messages to be understood correctly, thus raising the interest of potential volunteers.

Huang et al. (2016) also carried out a content analysis to examine behavior and strategies taken for channels such as Facebook by NGOs fighting diseases such as HIV/AIDS. In this way, NGOs perceive how their volunteers see them in terms of reputation, image, and recruitment. It was found that the reputation and image of NGOs in social networks that support social projects is positively identified by volunteers and members of non-profit organizations and institutions on digital channels such as Facebook.

Facebook, along with other social networks and digital platforms, led authors such as Liu and Lee (2010) to research how user information is used on social networks and web platforms by NGOs. The aim was to promote and improve collaboration between volunteers to achieve greater safety and acceptance of the proposed projects, concluding that the veracity of the information and the periodicity of publication of the contents was a key point in the development of NGO strategies. Guiang (2017) also investigated how followers’ participation on a digital platform can be increased, paying special attention to photo posts and content to improve the organization’s image so that the strongest impact can be made on users who follow these projects.

Therefore, there is no doubt about the interest of researchers in understanding how NGOs should communicate in digital environments and how to transmit messages effectively to volunteers to convey credibility and useful information. It has also been seen that the image, reputation, and visual identity variables of NGOs play a crucial role in the success of Internet communication and serve as motivation to users to support and become part of social projects.

## Hypothesis Development

It must be emphasized that trust in NGOs has been studied by Fast et al. (2013) and Demba et al. (2019). Their conclusions showed the importance of studying the confidence volunteers have in NGOs before becoming part of them and also when deciding to support their solidarity initiatives. Gefen et al. (2003) proposed the study of user behavior with NGO social proposals and investigated the perceived ease of use of websites and social networks on which NGOs promote their projects. It was suggested that these should be studied from the different perspectives of use and acceptance. Under this premise, we propose the following research hypothesis:

H1. *Trust in NGOs which use a web platform that promotes volunteering influences the perceived ease of use (PEOU) of the platform.*

Trust plays a crucial role in social sectors (Guiang, 2017). The organization of projects supported by different support organizations can increase the awareness of these initiatives and boost their image and reputation. Consequently, well-organized social NGO-type projects can improve the perceived usefulness of these projects, whether digital or offline, and considerably increase the number of interested volunteers (Dickinger and Stangl (2013). With these considerations in mind, the following hypothesis is proposed:

H2. *Trust in NGOs which use a web platform that promotes volunteering influences the perceived usefulness (PU) of that platform.*

The visual identity of NGOs is directly linked to the trust of the volunteers who support the social initiatives of such NGOs (Delone and McLean, 2003; Aladwani, 2006). If NGOs correctly manage their reputation and image strategies by improving their visual identity, the visual elements used should increase the trust that volunteers place in the social projects they choose to support (Aladwani, 2006; Demba et al., 2019). Using these ideas, we propose the following hypothesis:

H3. *The visual identity (IV) of NGOs which use a web platform that promotes volunteering influences the trust of platform users.*

Aladwani (2006); Delone and McLean (2003), and Dickinger and Stangl (2013) showed that the strategic image of organizations and the visual and multimedia elements the brand uses can define the characteristics and values of an organization. The TAM was extended to incorporate these ideas and measure the acceptance of a website based not only on its multimedia characteristics but also on those qualities that make up and define the visual identity of the company or organization. The IV construct measures the relationship between this variable and the perception of usefulness of a platform that NGOs use when in search of potential volunteers. The following hypothesis was therefore proposed:

H4. *The IV of NGOs influences the PU of a web platform that promotes volunteering.*

The visual identity of NGOs and the channels they use to promote their purposes has previously been studied. Likewise, the factors that make up the visual identity of the organizations link their image and reputation with the use of their websites (Aladwani, 2006). Studying the visual identity of NGOs can show if there is a significant link between the digital reputation of a web platform to support volunteering and the volunteers recruited. Using these ideas, hypothesis 5 was proposed:

H5. *The IV of NGOs influences the online image and reputation (IM) of a web platform that* promotes *volunteering.*

The set of sub-dimensions presented by Aladwani (2006) shows that the acceptance of online platforms needs to be studied because of the issues relating to the image and identity of organizations. The image and reputation of companies may also be one of the reasons for how users perceive the usefulness of social network technologies (Thongpapanl and Ashraf, 2011). Therefore, measuring the image and reputation of NGOs and how this may affect the acceptance of a technology from its perceived usefulness is an interesting area of research (Estriegana et al., 2019). Thus, the following hypothesis was proposed:

H6. *The IM of NGOs influences the PU of a social network that promotes volunteering.*

Aladwani (2006) explained the need to study the image and reputation of organizations from their website contributions, extending the TAM to measure the acceptance of a website (Revythi and Tselios, 2019). The study provided different variables that allowed users to identify the organizations that represent them due to the image and reputation shown, such as the type of content, messages, and information provided. The study looked at comprising features, transparency, and clarity. The model proposed by Aladwani (2006) and Dickinger and Stangl (2013) was used to define the construct that measures the acceptance of a platform that NGOs use from the image and reputation projected. How potential volunteers perceive the usefulness of this platform was also studied. Therefore, the following hypothesis was proposed:

H7. *The IM* of *NGOs influences the PEOU of a social network that promotes volunteering.*

The perceived ease of use of web platforms can make users decide to use them, as shown from the results presented by Toufaily et al. (2013). The perceived value of a technological product is also a key factor in the users’ decision to use it (Choi, 2019). Therefore, the importance of the ease of use of web pages and new online platforms must influence both user experience improvement and utility strategies. This will lead to users wanting to use the web platforms and spend more time connected to them (Demba et al., 2019). Hypothesis 7 was formulated after considering these points:

H8. *PEOU of a web platform that promotes volunteering influences PU.*

Zaitul et al. (2018) demonstrated the influence of perceived ease of use of web pages and discovered that the preconceived idea of users using a website is directly linked to their attitude about using it. Hart and Sutcliffe (2019) also linked the attitude about a website’s use to the influence of the medium or channel used to share information, such as social networks, websites, or applications. From these considerations, the following hypothesis was proposed:

H9. *PEOU of a web platform that promotes volunteering influences attitude toward using it.*

non-profit-making organizations explain their projects and promote social actions on different channels. One of these channels is the Internet, where web pages are a source of data that give credibility to initiatives (Kingston and Stam, 2013). The credibility of the information given may help users to positively perceive the proposed action (Stuart et al., 2014). Therefore, the quality and veracity of the information can influence users’ attitudes toward the use of digital platforms on which NGOs provide information about their projects (Fast et al., 2013). The following hypothesis was therefore proposed:

H10. *PU of a web platform that promotes volunteering influences attitude toward using it.*

It must also be understood that attitude toward the use of web platforms and how they are perceived by users influences the intention to use these digital platforms (Dickinger and Stangl, 2013). The attitude toward the use of a new website must be motivated with a positive intention to use it, which is gained from the perceived value of using it (Guriting and Oly Ndubisi, 2006). The attitude toward using a web platform that promotes volunteering should therefore also be studied taking into account that users should find the NGO’s projects interesting for them, and therefore, this interest is extended to the intention to use the technology itself (Scherer et al., 2019). Therefore, the following hypothesis was proposed:

H11. *Attitude toward using a web platform that promotes volunteering for NGO projects influences intention to use the platform.*

The perception of information as useful is important for it to be considered interesting on digital platforms (Saura et al., 2019b). This is especially true for the information given by NGOs about their projects in order to attract potential volunteers and share their achievements and new initiatives (Parsons and Woods-Ballard, 2003). Acceptance of web platforms that promote NGO projects is shown by the intention to use them by volunteers, who want to obtain quality information and decide whether or not to support such projects (Aladwani, 2006). The perceived usefulness and intention to use these platforms should therefore be analyzed. The following hypothesis was therefore proposed:

H12. *PU of a web platform that promotes volunteering influences intention to use it.*

The intention to use any piece of digital technology determines the actions that users take on social networks, web pages, or any other type of digital format in which there is an interaction between companies or organizations and users (Gefen et al., 2003; Aggelidis and Chatzoglou, 2019). Volunteers’ use of a social platform can increase intention to use it and vice versa (Revythi and Tselios, 2019). These actions can be measured to identify the influence and importance that intention to use has on the users’ actions (Aladwani and Palvia, 2002). The following hypothesis was therefore proposed:

H13. *The intention to use a web platform that promotes volunteering influences the use that volunteers make of this platform.*

## Methodology

### Measures

Technology Acceptance Model (Davis, 1989) has been one of the most widely used technology acceptance models in research to measure user behavior and acceptance of new technologies. The TAM consists of the relationships between different variables. For example, two primary variables that influence the intention of individuals to use technology are analyzed. One of them is perceived ease of use (PEOU, which measures an individual’s belief that using one particular technology is effort-free (Davis et al., 1989). TAM also contains a variable for perceived usefulness (PU), which is a user’s belief that using a particular piece of technology will improve the user’s performance (Koufaris, 2002). In addition, the attitude toward using (ATU) variable measures the positive or negative feelings a user may have for the use of any given technology.

In addition, TAM has the intention to use (UI) variable, which measures the user’s intentions to use the proposed technology. In this research, we increase the number of variables used in the TAM with variables for the image and reputation (IM) and visual identity (IV) of the NGO (Moon and Kim, 2001) following the findings of Gefen et al. (2003) in their study, which showed that the original TAM does not capture all the factors that affect website acceptance. In this study, IM refers to the public image and reputation of NGOs that may influence the acceptance of platforms or projects that are part of it. IV measures users’ perceived belief in the values that NGOs convey with their visual identity policies.

The trust (T) construct has also been added, which is a commonly used variable when extending the TAM (Gefen et al., 2003; Fast et al., 2013; Demba et al., 2019) and in this study represents the trust that volunteers have in the use and acceptance of a platform. The use online volunteer platform (UOVP) variable was also added, which measures volunteers’ use of an online volunteer platform following the research by Gefen et al. (2003); Huang et al. (2016), and Cheng (2020), which measures user behavior with NGOs digital platforms.

**FIGURE 1:**
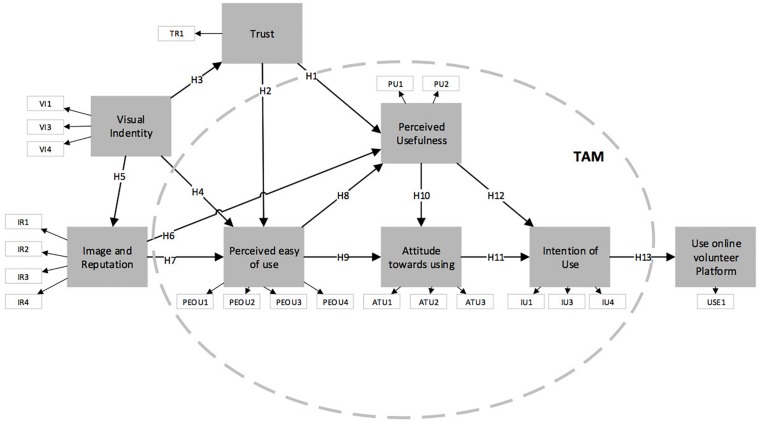
Proposed model.

The proposed hypotheses are all based on the information from different authors found in the literature review. They are all used in the extended TAM theoretical framework (Davis, 1989; Davis et al., 1992). The items contained within the discontinuous line in Figure 1 show the constructs: PU (2), PEOU (4), ATU (3), and UI (3). The constructs added to the model were as follows: IV (3) measured aspects such as the visual identity (logo, colors, typography) as a guiding factor for the quality of service of a non-profit-making organization and the ability to recruit volunteers using this visual identity. One of the items omitted in the statistical validation stage was the ability to opt out of collaborating on a website because its design does not please the user. The other construct was trust (2). The items used were the increase of trust in the non-profit-making organization and the feeling of greater safety on websites whose design seems appropriate. Another construct used was IM (4), which gave a measure of how the services of an organization were rated from its brand image, the reliability of the image of the organization, how easy it was to remember the organization from its logo and color, and finally, the importance of the website design. Finally, the use of the platform is measured with the capacity of the promoting platform to put volunteers and non-profit-making organizations in contact and increasing the number of volunteers. This second item was finally omitted following the results presented by Carmines and Zeller (1979), λ ≥ 0.707. They indicated that the commonality of an indicator (λ^2^) represents how much of the variation of an item is explained by the construct.

Likewise, the questionnaire items were measured with a Likert scale with values from 5, total agreement, to 1, total disagreement. Table 1 shows the questionnaire questions. Of these, for reasons of significance and statistical validity, eight were omitted, which are indicated without path coefficients.

**TABLE 1:** Measurement items.

Constructs	Items	Path coefficients (β)
Use online volunteer platforms Gefen et al. (2003) Huang et al. (2016); Cheng (2020)	USE1 [Using a social media platform that helps connect volunteers and non-profit associations would increase my desire to participate in such partnerships.]	1.000
	USE2 [The existence of this platform would increase the number of volunteers.]	Omitted
Attitude toward using Hart and Sutcliffe (2019)	AU1 [Its use would be positive for my life.]	0.927
	AU2 [Its use would be beneficial to my family and circle of friends.]	0.915
	AU3 [Its use would be beneficial to society.]	Omitted
Image and reputation online Aladwani (2006); Thongpapanl and Ashraf (2011)	IM1 [I value the service of an association based on its brand image.]	0.825
	IM2 [I consider that an organization with a proper brand image is more reliable.]	0.869
	IM3 [I remember an association more easily if I associate it with a logo and/or color.]	0.701
	IM4 [The design of an organizations website is of great importance to me.]	0.788
	IM5 [I would like brands to support the use of this platform.]	Omitted
Intention to use Aggelidis and Chatzoglou (2019) Revythi and Tselios (2019) Aladwani and Palvia (2002)	IU1 [I would be willing to search for and/or write volunteer offers on this platform.]	0.813
	IU2 [I would recommend using it to my family and friends.]	0.860
	IU3 [I hope to use this platform to easily volunteer in the coming months.]	0.854
	IU4 [I would accept advertising advice from this platform.]	Omitted
Visual identity Aladwani (2006); Delone and McLean (2003) Dickinger and Stangl (2013)	IV1 [Visual identity (logo, colors, typography.) gives me clues about the quality of an organization’s services.]	0.841
	IV2 [I consider more collaborating with a non-profit association when I like its visual identity.]	0.838
	IV3 [I consider more collaborating with a non-profit association when their visual identity is familiar to me.]	0.821
	IV4 [I gave up buying/collaborating on a website because I didn’t like its design.]	Omitted
Perceived ease of use Koufaris (2002) Fast et al. (2013)	PEOU1 [Its purpose is clear and understandable.]	0.744
	PEOU2 [I find it easy to access volunteer offers easily.]	0.821
	PEOU3 [The existence of such a platform would make it easier for me to be more concerned about social and/or environmental problems.]	0.723
	PEOU4 [I would find useful a platform where I could easily find how to help in those matters that really concern me.]	0.775
	PEOU5 [Learning how to use this website would be easy for me.]	Omitted
Perceived usefulness (PU) Gefen et al. (2003)	PU1 [Using this platform would make me feel better about myself.]	0.899
	PU2 [When using it, I expect to be helping society.]	0.908
	PU3 [The creation of such a platform would help the performance of non-profit associations participating on it.]	Omitted
Trust Gefen et al. (2003); Fast et al. (2013)Demba et al. (2019)	T1 [Contacting a non-profit association with this platform would make me more confident in that partnership.]	1.000
	T2 [I feel safer on websites whose design I think is appropriate.]	Omitted

The rest of the questionnaire questions were sociodemographic (gender, age, education level, and job). A question about the number of inhabitants in the city where the respondent lives and two questions about the subject studied at university were also included. These were: degree and form of collaboration with the non-profit-making organization and the name of the non-profit-making organization being collaborated with. These two points helped us to understand the characteristics of the sample being studied (see Table 2).

**TABLE 2:** Distribution of the sample, *n* = 254.

Items	Frequency	%
**Gender**		
Male	101	39.60
Female	153	60.00
Others	1	0.4
**Age**		
18–30 years old	155	60.8
31–45 years old	25	9.8
46–55 years old	45	17.6
56–65 years old	28	11.0
>65 years old	2	0.8
**Education level**	6.1.1	6.1.2
No studies	6	2.4
Diploma/advanced diploma	23	9.0
Bachelor’s degree	11	4.3
Professional qualification	24	9.4
Graduate university	191	74.9
**Job**		
Employed	1	0.4
Self-employed	10	9.1
Student	118	46.3
House work	9	3.5
Does not work (unemployed)	11	4.3
Does not work (other situations: retired)	14	5.5
**Degree and form of collaboration**		
Never	71	27.8
Yes, voluntarily	70	27.5
Yes, economically	67	26.3
Yes, financially and voluntary	47	18.4

The questionnaire was formatted electronically with Google Forms and distributed on social networks, email, and the university’s own online content platform.

### Data Collection and Sample

The sample size was *n* = 254 respondents. The sample was obtained with the collaboration of students taking the Business Administration degree course at Rey Juan Carlos University in Madrid and the Marketing degree at the University of Seville.

The distribution of the questionnaire took place between April and July 2019. The type of sampling was non-random and convenient, as the students themselves distributed it to acquaintances, friends, and family. The statistical distribution of the sample and its characteristics are detailed in Table 2.

Table 3 presents the associations and non-profit organizations with which the participants of this exploratory study collaborated.

**TABLE 3:** Organizations in which the sample participates.

Organization	Frequency	Number of participants
Unicef	38.8%	99
Cruz Roja	33.3%	85
Médicos sin Fronteras	22.4%	57
Manos Unidas	20.4%	52
Greenpeace	10.6%	27
Caritas	10.2%	26
Save the Children	9.4%	24
Unhcr	8.6%	22
Oxfam Intermon	5.1%	13
Aldeas Infantiles	3.9%	10
World Wide Fund for Nature	3.9%	10
Amnesty International	2.4%	6
Help in Action	2.0%	5
Asociación Española Contra El Cáncer	1.6%	4
Banco de Alimentos	1.6%	4
Ayuda Humanitarian	0.4%	1

**FIGURE 2:**
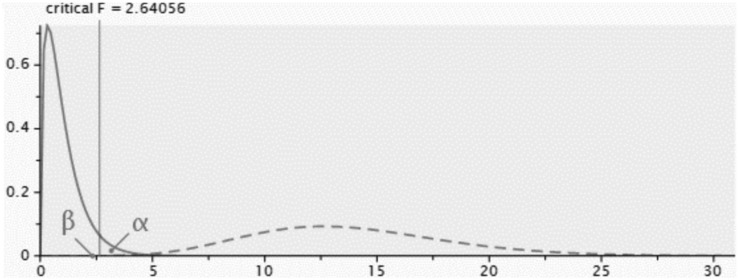
Central and non-central distributions. Calculation of the power. Source: G*Power (Faul et al., 2009).

To determine the minimum sample size for PLS modeling, Hair et al. (2014) recommend using the Cohen table. The G^∗^Power software (Faul et al., 2009) was used to make this calculation. Firstly, the count test of the dependent construct or the one with the highest number of predictors was carried out. In this study, these were PU and PEOU, which both had a score of 3. The following parameters were used for the calculation: the power of the test (power s 1 – error prob. II) and the effect size (*f*^2^). Cohen (1988) and Hair et al. (2014) recommend a power of 0.80 and average effect size *f*^2^ of 0.15. The number of predictors was 3, i.e., the constructs that establish causal relationships with either of the two constructs: PU and PEOU (see Figure 1). Using PLS with these constructs the minimum sample size to be used was 251. Figure 2 demonstrates the test result using the software.

Therefore, the minimum sample calculated for the example must be 251 cases. The data obtained gives a power of 99.98%, as shown in Figure 2. Following the research by MacKenzie et al. (2005), a model of reflective constructs was proposed, as shown in Figure 1.

## Data Analysis

First, an individual reliability analysis of the items was performed using the values for the loads. According to Carmines and Zeller (1979), the minimum level for acceptance as part of the construct was set at λ ≥ 0.707. The communality of a variable (λ^2^) shows the part of the variance that is explained by the factor or construct (Bollen, 1989). Therefore, a value of λ > 0.707 indicates that each measure represents at least 50% (0.707 ≥ 0.5) of the variance of the underlying construct (Henseler et al., 2009). Those indicators that did not reach that minimum were removed (Barclay et al., 1995). The results of the measurement model are shown in Table 1.

Subsequently, an analysis of the internal consistency was carried out. Traditionally, Cronbach’s alpha is analyzed to test the consistency of a construct, assuming that all indicators are equally reliable, meaning that they all have the same loads in the construct (Hair et al., 2014) and present values between 0 and 1. The lower limit for accepting construct reliability is usually set to between 0.6 and 0.7 (Hair et al., 2005).

The greatest validity will be with values close to 1. However, composite reliability (CR) measures the consistency of a construct based on its indicators (Götz et al., 2010), i.e., the rigor with which these items are measuring the same latent variable, assuming that CR > 0.7 (exploratory study), CR > 0.8 (advanced research), and CR < 0.6 (lack reliability). In our case, CR > 0.8, so we are can consider this an advanced research study (Henseler et al., 2009; MacKenzie et al., 2011).

The most common measure for assessing convergent validity in PLS-SEM is average variance extracted (AVE). Using the same basis as that used with individual indicators, an AVE value of 50% or higher means that, on average, the construct explains more than half of the variance of its own indicators (Fornell and Larcker, 1981; Hair et al., 2014). As seen in Table 4, all indicators reached this lower limit. Finally, another indicator, known as rho_A, has also been included in Table 4 (Dijkstra and Henseler, 2015a, b), and all the constructs exceed the value 0.7.

**TABLE 4:** Constructs and their measurement items.

	Cronbach’s alpha	rho_A	Composite reliability	Average variance extracted (AVE)
Attitude toward using	0.822	0.825	0.918	0.849
Image and reputation online	0.807	0.814	0.875	0.637
Intention of use	0.88	0.883	0.917	0.735
Visual identity	0.786	0.813	0.872	0.694
Perceived ease of use	0.776	0.775	0.856	0.598
Perceived usefulness	0.775	0.776	0.899	0.816
Trust	1	1	1	1
Use online volunteer platforms	1	1	1	1

Table 5 shows the correlations between the constructs. A construct should share more variance with its measurements or indicators than with other constructs in a given model (Henseler et al., 2009). To check this, we need to see if the square root of the AVE (in bold in Table 5) is greater than the correlation between the construct and the other constructs in the model. The square roots of the AVE can be found on the diagonal of Table 5. In our study, this condition is met by all latent variables.

**TABLE 5:** Correlations between constructs (Fornell and Larcker, 1981).

	ATU	IR	IU	VI	PEOU	PU	T	USE
Attitude toward using (ATU)	0.921	11.1	11.2	11.3	11.4	11.5	11.6	11.7
Image and reputation online (IR)	0.294	0.798	11.8	11.9	11.10	11.11	11.12	11.13
Intention of use (IU)	0.763	0.331	0.857	11.14	11.15	11.16	11.17	11.18
Visual identity (VI)	0.317	0.709	0.314	0.833	11.19	11.20	11.21	11.22
Perceived ease of use (PEOU)	0.617	0.395	0.746	0.308	0.774	11.23	11.24	11.25
Perceived usefulness (PU)	0.643	0.348	0.696	0.295	0.695	0.904	11.26	11.27
Trust (T)	0.461	0.346	0.543	0.313	0.666	0.567	1	11.28
Use online volunteer platform (USE)	0.534	0.236	0.605	•	•	•	•	•

It can therefore be said that constructs share more variance with their indicators than with other constructs of the investigated model (Henseler et al., 2009) and are valid on the basis of this first analysis. Nonetheless, Henseler et al. (2015) worked on simulation studies that showed that the lack of discriminatory validity is best detected by another technique, which is the heterotrait–monotrait ratio (HTMT). Table 6 shows the results obtained. All the HTMT ratios for each pair of factors is <0.90 (Gold et al., 2001).

**TABLE 6:** Heterotrait–monotrait (HTMT) ratio.

	ATU	IR	IU	VI	PEOU	PU	T	USE
Attitude toward using	11.28.1	11.28.2	11.28.3	11.28.4	11.28.5	11.28.6	11.28.7	11.28.8
Image and reputation online	0.358	11.29	11.30	11.31	11.32	11.33	11.34	11.35
Intention of use	0.897	0.391	11.36	11.37	11.38	11.39	11.40	11.41
Visual identity	0.383	0.857	0.364	11.42	11.43	11.44	11.45	11.46
Perceived ease of use	0.768	0.495	0.896	0.373	11.47	11.48	11.49	11.50
Perceived usefulness	0.804	0.438	0.838	0.366	0.891	11.51	11.52	11.53
Trust	0.51	0.385	0.577	0.339	0.748	0.644	11.54	11.55
Use online volunteer platforms	0.587	0.26	0.643	0.226	0.764	0.599	0.418	11.56

**FIGURE 3:**
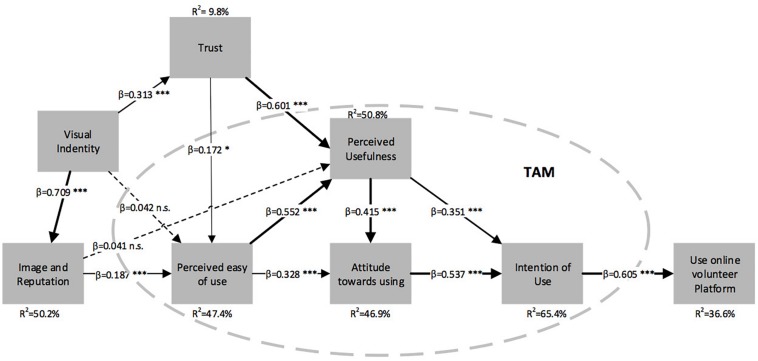
Quality of the measurement model and the structural model.

Table 7 and Figure 3 show the findings for the formulated hypotheses. The tests carried out reveal that *R*^2^ = 9.8% of the variance in T is explained by the IV construct. IV also explains the variance of the IM construct (*R*^2^ = 50.2%). The explanatory capacity of this construct is almost the highest of the model and is only surpassed by tenths by PU (*R*^2^ = 50.8% of the variance). It is also worth noting that *R*^2^ = 65.4% for the variance of the UI. This corroborates the use of the theoretical TAM framework for the study.

**TABLE 7:** Comparison of the hypotheses.

Number	Hypothesis	Path coef. (β)	*t*-Statistic (β/STDEV)	*P*-value	Supported
H1	Trust → perceived ease of use	0.601	12.053	0	Yes
H2	Trust → perceived usefulness	0.172	2.306	0.021	Yes
H3	Visual identity → trust	0.313	4.911	0	Yes
H4	Visual identity → perceived usefulness	0.042	0.643	0.520	No
H5	Visual identity → image and reputation online	0.709	22.031	0	Yes
H6	Image and reputation online → perceived usefulness	0.041	0.621	0.535	No
H7	Image and reputation online → perceived ease of use	0.187	3.357	0.001	Yes
H8	Perceived ease of use → perceived usefulness	0.552	8.132	0	Yes
H9	Perceived ease of use → attitude toward using	0.328	4.78	0	Yes
H10	Perceived usefulness → attitude toward using	0.415	6.034	0	Yes
H11	Attitude toward using → intention to use	0.537	8.716	0	Yes
H12	Perceived usefulness → intention of use	0.351	5.194	0	Yes
H13	Intention to use → use online volunteer platforms	0.605	12.742	0	Yes

The explanatory capacity of the model as a whole can be measured by studying the *R*^2^ value of the dependent variable UOVP. This had a total variance of *R*^2^ = 36.6%. Figure 3 shows the explanatory capabilities of the other constructs. In their research, Hair et al. (2014) set the cut-off points for the relevant, moderate, and weak levels at R^2^ = 0.75, 0.50, and 0.25. It can therefore be seen that this model has an explanatory capacity between moderate and weak for online volunteering platforms with non-profit associations.

On the other hand, comparing the hypotheses shows that 11 hypotheses are supported and 2 are not. Table 7 shows the different *p*-values of each. It can be seen that the relationships with the highest path coefficient are H5, IV with online IM, which is significant (β = 0.709, *p*-value = 0.000); H13, UI with UOVP (β = 0.605, *p*-value = 0.000); H12, UI with UOVP (β = 0.605, *p*-value = 0.000); and H1, T with PEOU (β = 0.601, *p*-value = 0.000). All these hypotheses, along with H3, H5, H8, H9, H10, H11, and H12, were supported with a 99.9% confidence level.

The ratios with the lowest path coefficient turned out to be H7, online IM with PEOU (β = 0.187, *p*-value = 0.001), with a 99% confidence level, and H2, T with PU (β = 0.172, *p*-value = 0.021), with a 95% confidence level. The unsupported hypotheses were H4, IV with PU (β = 0.042, *p*-value = 0.520), and H6, online IM with PU (β = 0.041, *p*-value = 0.535). This means that the PU is not affected by the IV and the online IM, with a 95% confidence.

A blindfolding procedure was then used to omit part of the data of a construct during parameter estimation, and then, the estimated parameters were used to estimate the originally omitted data (Chin, 1998). In this way, it was possible to study the predictive level of the model by using the Stone–Geisser (*Q*^2^) test (Geisser, 1974; Stone, 1974). The model was found to be predictive (*Q*^2^ = 0.352), as *Q*^2^ > 0.

The measurement of the approximate adjustment of the model (Henseler et al., 2016; Henseler, 2017) is found from the standardized root mean square residual (SRMR) (Hu and Bentler, 1998, 1999), which measures the difference between the observed correlation matrix and the correlation matrix implied by the model. The SRMR therefore shows the average magnitude of the differences. This means that a lower SRMR shows a better fit. In our case, SRMR = 0.071, which follows the recommendation that states that a model has a good fit when SRMR < 0.08 (Hu and Bentler, 1998).

## Discussion

The results of this study indicate that extended TAM is an appropriate model for the analysis of technological acceptance of social platforms that allow volunteers or other professionals to be recruited. As indicated before, TAM was extended with IM, IV, and T variables in this study. The model used in the research presents a good *R*^2^ value for both the intention of use of the platform, which explains 65.4% of the variance, and the use of the platform, which explains 36.6% of the variance.

An NGO’s online IM and IV constructs were found to have no direct effect on PU. In the literature, it is usually found that the appearance of organizations—iconic visual elements such as logos, colors, typography, and multimedia linked to the brand—helps define their characteristics and values, at least for commercial companies (Delone and McLean, 2003; Dickinger and Stangl, 2013). However, this study has shown that the online identity and reputation of NGOs as well as the visual identity elements (such as images or logos) do not have a direct effect on how users and volunteers support these platforms, thus obtaining results different from those found by Aladwani (2006) in the professional as opposed to the social industry. This finding is an important contribution to literature, as NGOs should focus on the message and the contents that accompany their initiatives, instead of focusing on the iconographic design and identity of their web pages or visual contents that are included in the messages they share on Internet (Maloney and Rosenthal, 2017).

However, IV is also usually related to the PU (Aladwani, 2006). The unusual results of this study may be due to the nature of the organizations that were analyzed. NGO volunteers are more interested in the values of the organization itself and the social causes that it defends, rather than on the visual image or the brand name. However, in a changing ecosystem in which new technologies and multimedia channels are constantly appearing, authors such as Cheng (2020) showed the importance of keeping users or volunteers motivated with projects that are seen as useful and that can create loyalty with long-term volunteers. It was also found that the quality of the message shared by NGOs, the values that make up these organizations (the causes that are supported, such as social, human, cultural, etc.) and the channel on which they are shared are the most important points for an NGO’s success in attracting volunteers who support their projects. These findings are in agreement with the results presented by Silva et al. (2018).

Therefore, coinciding with the results of Waters (2007), NGOs should pay special attention to the content and words that form the messages they share on digital platforms or social networks with users and volunteers. As concluded by Saura et al. (2019a), these messages can influence users in different ways about their intention of use and behavior when they interact with the content on digital ecosystems. Likewise, as shown in the results, the visual identity of the platforms that support social projects and seek volunteers positively affects the confidence that volunteers have in the use of such digital environments. This was also observed by Amichai-Hamburger et al. (2008). Moreover, the visual identity of the platform, although it does not influence the perceived utility by the volunteers, does affect the online image and reputation of the NGOs themselves. In other words, the positive reputation of a platform can influence the reputation of NGOs that agree to promote their social projects on it.

This fact is a contribution to the literature that coincides with the conclusions of authors such as Van Huijstee and Glasbergen (2010) and Boddewyn and Doh (2011), in which they profess that the combination of elements and business synergies can improve the online reputation of companies and organizations. It is important to consider the evidence and point out the positive relationship found between the UI and UOVP, which justifies using the type of platform that promotes NGO projects and raises interest in them. Once volunteers know the services that a platform offers and intend to continue using it, they usually register their user profiles, as it is considered a useful and effective way to continue helping NGOs and their projects (Emrich et al., 2014).

Finally, UI has a positive and direct relationship with the UOVP construct. This is a classic relationship in the literature about the Theory of Reasoned Action (Fishbein and Ajzen, 1980), the technology acceptance model TAM (Davis, 1989), and Unified theory of acceptance and use of technology (UTAUT) (Venkatesh et al., 2003). This study confirms this direct and positive relationship. The greater intention of volunteers to seek volunteer offers and recommend the platform to their friends and acquaintances is the interpretation of the use of the platform.

## Conclusion

The aim of this work was to analyze the acceptance of a platform that allows NGOs to attract well-prepared and motivated potential young volunteers. Using the TAM, proposed by Davis (1989), a TAM was proposed that was extended and adapted for the recruitment of volunteers by the organizations. Examining the different elements that affect the adoption of potential volunteers with technology is extremely important for NGO managers. The recruitment of well-prepared and motivated young volunteers will have a greater likelihood of success with the modernization of the recruitment methods that are used (Saura et al., 2019c).

In this study, 11 of the proposed hypotheses were accepted, and 2 were rejected, as explained above. The relationship that explains H1 shows that trust in the NGO platform influences PEOU, which in turn shows that the strategy used to recruit volunteers should be based on ease of use with messages that transmit trust and faithfully exhibit the work of the NGO. Similarly, H2, which investigates the relationship of T and PU, has a positive result, which shows that trusting the NGO platform influences volunteers to register their online profile on the platform.

As stated above in the *Discussion* section, the IV construct influences the trust users have in a platform (H3). This shows the importance of the visual identity and the elements that make up the NGO platform in positively influencing volunteers’ trust in the platform and transmitting this trust to the visitors of the platform. The results for H4, however, did not show a strong relationship of IV with PU, which means that the NGOs and the platforms they use should communicate their values in their messages and communication strategies, instead of relying on elements such as logos and specially created images.

H5 investigated the relationship of IV with online IM and showed that an NGO that uses an Internet platform with good visual identity can improve the image and reputation it has with online users. H6, however, was shown to have a negative result, which means that the online IM does not influence PU. This result has been commented on above, and it was concluded that the image and reputation of an NGO’s platform does not affect its perceived usefulness, as the volunteers support the NGO and its projects. These projects are the elements that influence the volunteers to use the services supplied by a platform and not the platform itself (Saxton et al., 2014). H7 showed that there exists a positive relationship between online IM and PEOU. This means that the image and reputation of the platform can affect how easy volunteers who support an NGO and its projects feel it is to use the platform.

The relationship between PEOU and PU (H8) was shown to have a positive result, which confirms the influence of how easy to use a platform used by an NGO is believed to be when starting and promoting new projects on how useful these projects are believed to be. The relationships in H9, which studied the influence of PEOU and ATU, and H10, which investigated the influence of PU and ATU, were also confirmed. This showed how the perceived ease of use and usefulness of an NGO’s platform influenced the attitude of volunteers toward using a platform of this type. The positive relationships in H11 and H12 were verified and showed that ATU and PU both positively influenced UI. H13, as has been discussed above, shows how the intention to use a platform positively influences the use of the platform by volunteers who support the NGO. This shows that this type of platform must be used to encourage the interest of volunteers.

### Theoretical Implications

The theoretical implications of the research show that the proposed relationships were used to analyze the acceptance of platforms that promote NGO projects and their search for volunteers with constructs such as image and online reputation, visual identity, and online use of a volunteer platform. The results of this research can be used to improve and define efficient communication and marketing strategies for non-profit organizations using the theoretical and strategic results that have been found.

Likewise, it has been shown that strategic alliances in this ecosystem, using digital technologies such as social networks and the Internet, can help NGOs to express their values and projects effectively to volunteers. Platforms that promote social projects and need the support of volunteers and users who invest in their projects can use the results of this study to improve their presence on the Internet and expand their plans for digital communication and strategic alliances.

### Practical Implications

The results of this research show that the communication strategies for NGOs and platforms that rely on volunteers are of utmost importance for successful results on digital channels. Managers and executives of NGOs and public institutions that have non-profit-making projects or that provide communication campaigns to promote NGOs can use the results of this study to identify the importance of visual identity, online reputation, and the use of NGO platforms.

The results of this research can also be used by NGO managers to modify and extend the scope of their communication strategies in order to recruit more volunteers into their projects. The most important messages to be communicated must give importance to the values of the organization and not be centered on the graphical and design elements of the NGO.

The limitations of this research are due to the size of the test group and the profile of the questioned individuals. Also, the sample could be extended to potential volunteers of other ages, as this study is biased toward members of Generation Z and against other cultures and countries other than Spain. Finally, a longitudinal study would allow the verification, or not, of the relationships over time. In this study, the research was carried out with potential young volunteers who are used to using social media and platforms on the Internet. This is positive because a gap in the literature has been covered, although the theoretical basis of the variables was not applied to a wide enough range of participants for the relationships to be confirmed in all possible cases. It can be said, however, that the results of this study provide important contributions to the literature as a base for future investigations into NGOs and volunteer recruitment projects on digital environments.

## Data Availability Statement

The raw data supporting the conclusions of this article will be made available by the authors, without undue reservation, to any qualified researcher.

## Ethics Statment

Ethical review and approval was not required for the study on human participants in accordance with the local legislation and institutional requirements. Written informed consent from the participants was not required to participate in this study in accordance with the national legislation and the institutional requirements.

## Author Contributions

All authors listed have made a substantial, direct, and intellectual contribution to the work, and approved it for publication.

## Conflict of Interest

The authors declare that the research was conducted in the absence of any commercial or financial relationships that could be construed as a potential conflict of interest.
